# A novel myopathy with autophagic vacuoles associated with biallelic variants in *CLN8*

**DOI:** 10.1111/bpa.70128

**Published:** 2026-07-26

**Authors:** Ulrika Lindgren, Carola Hedberg‐Oldfors, Kittichate Visuttijai, Sara Nordström, Hans Goebel, Anders Oldfors

**Affiliations:** ^1^ Department of Laboratory Medicine, Institute of Biomedicine, Sahlgrenska Academy University of Gothenburg Gothenburg Sweden; ^2^ Department of Clinical Pathology Sahlgrenska University Hospital Gothenburg Region Västra Götaland Sweden; ^3^ Neuromuscular Center, Department of Neurology Sahlgrenska University Hospital Gothenburg Region Västra Götaland Sweden; ^4^ Department of Clinical Genetics and Genomics Sahlgrenska University Hospital Gothenburg Region Västra Götaland Sweden; ^5^ Department of Clinical Neuroscience, Institute of Neuroscience and Physiology, Sahlgrenska Academy University of Gothenburg Gothenburg Sweden; ^6^ Department of Neuropathology Charité—Universitätsmedizin Berlin Berlin Germany

**Keywords:** CLN8, epilepsy, myopathy, neuronal ceroid lipofuscinosis

## Abstract

Autophagic vacuoles in muscle fibers are a characteristic finding in several muscle diseases and usually indicate perturbed lysosomal protein degradation. Some of these are associated with defects in proteins directly involved in autophagy and lysosomal degradation. The gene *CLN8* encodes an endoplasmic reticulum transmembrane protein, previously associated with childhood‐onset neuronal ceroid lipofuscinosis (NCL), a group of lysosomal storage diseases. We describe the clinical features and results from pathology, genetic, and proteomic investigations in an adult‐onset myopathy with autophagic vacuoles associated with biallelic variants in *CLN8*. A 40‐year‐old woman presented with seizures followed by transient muscle weakness and myalgia. Creatine kinase and myoglobin levels were moderately elevated. Over time, she developed progressive muscle weakness and cognitive fatigue. Muscle biopsy showed an autophagic vacuolar myopathy with fat tissue replacement and increased interstitial connective tissue. There was a marked immunohistochemical increase of markers of autophagy such as lysosomal‐associated membrane protein 2 (LAMP2), microtubule‐associated protein 1A/1B‐light chain 3 (LC3), and sequestosome1/p62, as well as lysosomal deposition of curvilinear‐like, autofluorescent material containing subunit c of mitochondrial adenosine triphosphate (ATP) synthase (mitochondrial ATP synthase membrane subunit c locus 3 [ATP5MC3/SCMAS]), typical for some forms of NCLs, including CLN8. Blood lymphocytes showed typical fingerprint inclusions. Genetic analysis revealed biallelic *CLN8* variants, c.511C>T; p.P171S and c.536T>A; p.L179H. Proteomic analysis demonstrated upregulation of proteins involved in autophagy, muscle regeneration, and protein turnover. Proteins associated with oxidative phosphorylation were downregulated, except for ATP5MC3/SCMAS, which showed accumulation. In conclusion, we describe a novel myopathy with autophagic vacuoles and characteristic features of ceroid lipofuscinosis, including autophagosomal/lysosomal deposition of curvilinear‐like, autofluorescent material containing ATP5MC3/SCMAS. This disease appears to be an unusual adult‐onset form of CLN8.

## Graphical abstract

We describe a novel adult‐onset myopathy with autophagic vacuoles and characteristic features of ceroid lipofuscinosis associated with biallelic *CLN8* variants, seizures, and muscle weakness. Autophagosomal/lysosomal deposition of curvilinear, autofluorescent material containing the mitochondrial adenosine triphosphate (ATP) synthase membrane subunit c locus 3/subunit c of mitochondrial ATP synthase (ATP5MC3/SCMAS) protein and an increase of autophagic markers in muscle tissue is shown by combined diagnostic methods.

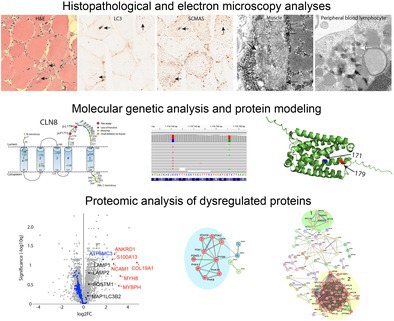

## 1 INTRODUCTION

Autophagic vacuoles in muscle fibers are a characteristic finding in several heterogeneous groups of muscle diseases referred to as hereditary or acquired protein aggregate myopathies (PAM) [1, 2], hereditary myofibrillar myopathies (MFM) [3, 4], autophagic vacuolar myopathies [5, 6], and multisystem proteinopathies (MSP) with muscle involvement [7], which are different but partially overlapping entities. Autophagic vacuoles are usually marked by microtubule‐associated protein 1A/1B‐light chain 3 (LC3), lysosomal‐associated membrane protein 2 (LAMP2), and sequestosome 1 (SQSTM1/p62), and they generally indicate disturbed autophagic flux or lysosomal protein degradation.

The neuronal ceroid lipofuscinoses (NCLs) comprise a group of clinically and genetically heterogeneous neurodegenerative disorders defined by the accumulation of lipofuscin (ceroid) in tissue and loss of neurons. Typical symptoms include seizures, cognitive impairment, and loss of vision. The gene *CLN8* on chromosome 8 encodes an endoplasmic reticulum (ER) transmembrane protein involved in transfer of lysosomal enzymes [8]. Biallelic variants in *CLN8* have been associated with NCL. Northern Epilepsy or progressive epilepsy with mental retardation (EPMR) [9, 10], has been described in Finland among related families all carrying a homozygous Arg42Gly missense variant in *CLN8*, with onset of therapy‐resistant seizures at 5–10 years of age followed by a progressive decline of cognitive skills. Another form is the Turkish variant of late‐infantile NCL (v‐LINCL), with onset at 2–7 years of age and a more severe course [11]. New pathogenic *CLN8* variants are continuously described [12] and more than 50 pathogenic *CLN8* variants have been reported to be associated with NCL.

In this study, we describe the clinical features, pathology, genetics, and proteomics of a novel myopathy with prominent autophagic vacuoles associated with biallelic *CLN8* variants in the first reported individual with adult‐onset neuronal ceroid lipofucinosis linked to *CLN8*.

## 2 MATERIALS AND METHODS

### 2.1 Muscle biopsy and morphological investigation

Skeletal muscle biopsy from the quadriceps muscle, vastus lateralis, was frozen in isopentane cooled in dry ice and processed for analysis using standard techniques, including histochemistry, enzyme histochemistry, and immunohistochemistry (Tables S1 and S2) [13]. Muscle fiber typing was based on myosin heavy chain (MyHC) expression using immunofluorescence as described [14]. For electron microscopy, a muscle specimen and peripheral blood lymphocytes (PBLs) were fixed in glutaraldehyde and processed essentially as described [13].

### 2.2 Genetic analysis

Total genomic DNA was isolated from muscle biopsy specimens using standard protocols. The DNA was subjected to whole genome sequencing (WGS) according to manufacturer's protocols (Illumina, San Diego, CA, USA). The paired‐end reads were aligned to the reference genome (hg19). Variants were called and filtered for identification of potentially pathogenic variants in candidate genes associated with NCLs listed as disease/gene symbol (CLN1/*PPT1*, CLN2/*TPP1*, CLN3/*CLN3*, CLN4/*CLN4*, CLN5/*CLN5*, CLN6/*CLN6*, CLN7/*CLN7*, CLN8/*CLN8*, CLN10/*CLN10*, CLN11/*GRN*, CLN12/*ATP13A2*, CLN13/*CTSF*, CLN14/*KCTD7*, CLN15/*CLCN6*) and myopathy based on The Gene Table of Neuromuscular Disorders 2024 (www.musclegenetable.fr/) [15].

### 2.3 Proteomic investigation

Skeletal muscle protein extracts from the patient and eight normal controls were prepared from fresh frozen muscle biopsies. For quantitative analysis the proteins were labeled using TMTpro 18‐plex isobaric mass tagging reagents (Thermo Fisher Scientific) and analyzed by nanoscale liquid chromatography–tandem mass spectrometry (LC‐MS^3^) as previously described [16]. Raw files were processed and analyzed with Proteome Discoverer against UniProt Swiss‐Prot Homo Sapiens using Sequest as a search engine. The gene symbols are used in tables and figures to describe the encoded proteins. Protein data were partly analyzed with the software Omics Playground (BigOmicsAnalytics, v4.1.2) [17]. To identify differentially expressed proteins, the data were log2‐transformed, and then, for each protein, log2 fold change (log2FC) and *p*‐values were computed using *t*‐test for patient versus controls. Additional pathway and protein–protein interaction analyses were also performed using different search tools and web resources, such as the STRING website (https://string-db.org/) [18]. In the STRING analysis 139 proteins were included based on false discovery rate (FDR) = 0.2; Log2FC <−1 or >1 (Table S3).

### 2.4 Controls

Eight age‐matched individuals with normal muscle biopsies who had been investigated for a possible mitochondrial disease or inflammatory myopathy served as controls for muscle immunohistochemistry and mass spectrometry. Muscle disease had been excluded by clinical, biochemical, and pathological investigations.

## 3 RESULTS

### 3.1 Case report

A 40‐year‐old woman presented with a focal to bilateral tonic–clonic seizure triggered by computer screen exposure. She had normal motor development and, apart from transient mental fatigue and hereditary otosclerosis, she was previously healthy. Family history was unremarkable for epilepsy or neuromuscular disorders; one sister had a history of myelitis in her thirties, and the other reported transient fatigue in her twenties. Brain magnetic resonance tomography (MRI), electroencephalography (EEG) with photic stimulation, and laboratory investigations were all considered to be normal.

Over the following years, the patient developed fluctuating fatigue, diffuse muscle weakness, and myalgia affecting the hips, back, and legs, along with increased photosensitivity. A second seizure occurred at age 43 with focal onset evolving to bilateral tonic–clonic, again triggered by computer screen exposure. Neurological examination and EEG (without photic stimulation) were normal. She was diagnosed with epilepsy and initially treated with carbamazepine, later switched to lamotrigine. Because of increased back pain after initiation of lamotrigine, treatment was changed to lacosamide, which relieved the pain but was associated with recurrence of seizures during computer use. Fatigue and muscle symptoms worsened after each seizure, followed by gradual recovery. Addition of brivaracetam resulted in adequate seizure control.

At 47 years of age, she had eczema‐like skin changes over the knuckles, and she was assessed at the dermatology and rheumatology clinics. Her neck flexors were slightly weak. Creatine kinase (CK) was 18 μkat/L (reference 0.6–3.5 μkat/L), myoglobin 185 μg/L (reference <90 μg/L), and alanine transaminase (ALT) 1.0 μkat/L (reference 0.15–0.75 μkat/L). Muscle biopsy was performed on the suspicion of dermatomyositis.

**FIGURE 1:**
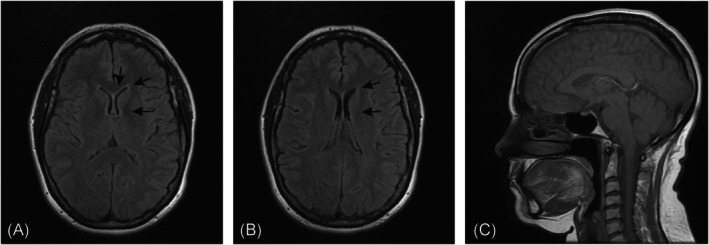
Magnetic resonance tomography of the brain at 48 years of age. (A and B) Bilateral symmetric discrete and diffuse enhanced signal in periventricular white matter (arrows) (T2/FLAIR). (C) Sulci appear normal and no atrophy is seen (T1).

After muscle biopsy, the patient was reassessed at the neuromuscular clinic. Neurological examination, including limb muscle strength and cranial nerves, was normal. Repeat brain MRI showed a subtle non‐specific bilateral symmetrically increased T2/FLAIR signal in periventricular white matter consistent with NCL, which, in retrospect, was also present in the first MRI (Figure 1A–C). Neuropsychiatric examination showed a possible decline of attention, language, memory, and executive functions, and signs of anxiety and depression. Eye examination showed small pigmental accumulations in the macula and a possible diffuse central photoreceptor layer but was otherwise normal. Electroretinogram was not performed, nor repeated EEG with photic stimulation.

Over the next years, the fatigue became more pronounced, causing her to work shorter days and need more rest. Muscle weakness progressed, and at 50 years of age she had moderate weakness in elbow flexion (57%) and extension (50%), as well as hip flexion (74%) (percentages describe her mean strength measured with myometer using the MAKE‐ or BREAK‐methods compared to the age‐ and sex‐matched reference value) [19, 20]. Both shoulder abduction (right 57%, left 51%) and knee extension (right 74%, left 59%) showed asymmetric moderate weakness. Forced vital capacity was mildly decreased (80% of the reference value) and she described an exertional shortness of breath. Her 6‐min walk test had decreased from a normal value at 49 years of age to 73% of the reference value. Grip strength was normal. She still has no seizure manifestations using a combined treatment of lacosamide and brivaracetam. CK, myoglobin, and liver enzymes are continuously slightly to moderately elevated.

### 3.2 Morphological analysis of skeletal muscle and PBLs

**FIGURE 2:**
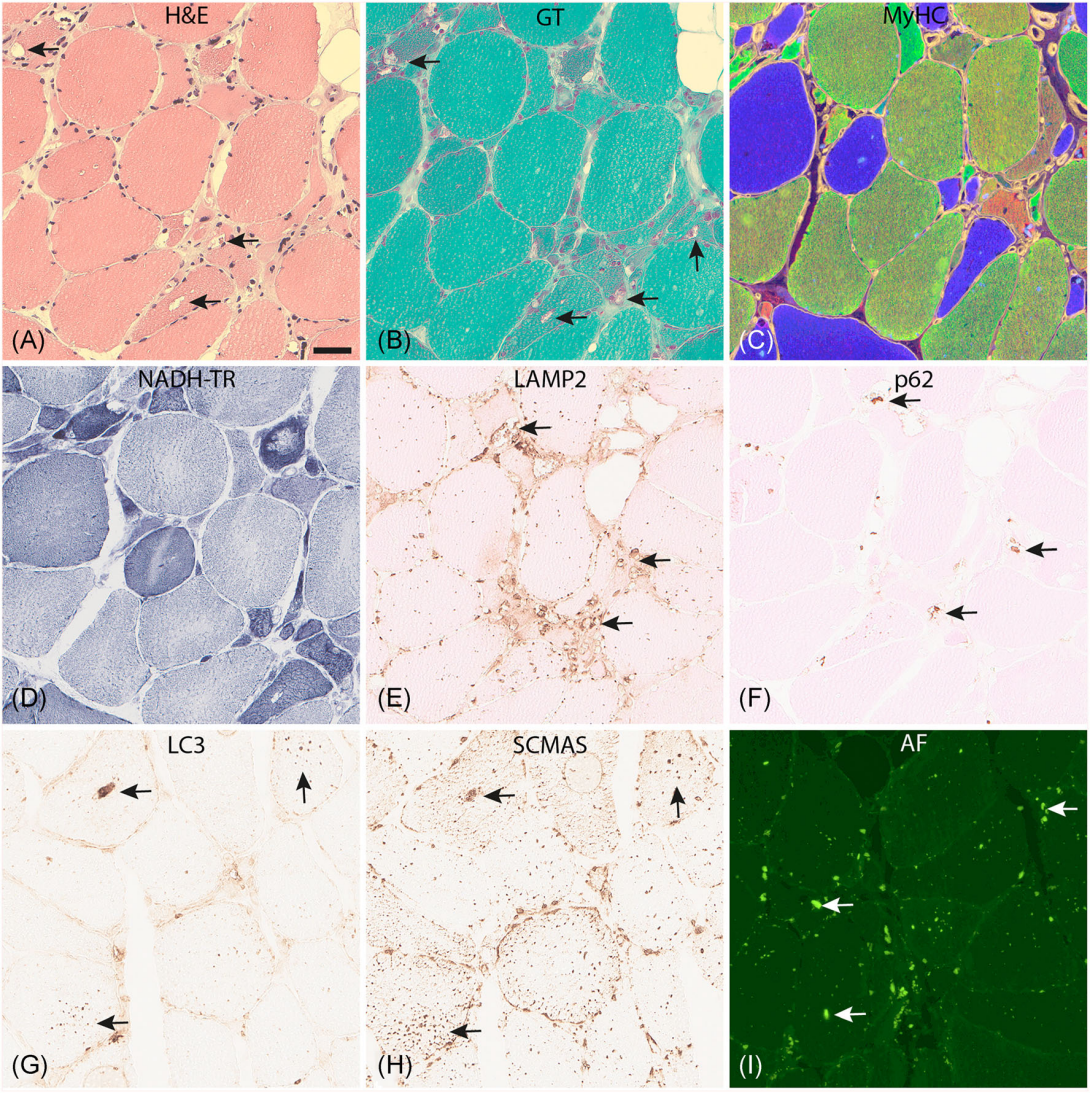
Histopathological analysis of the vastus lateralis muscle. (A and B) Vacuolated atrophic muscle fibers, hypertrophic muscle fibers, fat tissue replacement and increased interstitial connective tissue. Several rimmed vacuoles are present (arrows) (Hematoxylin and eosin [H&E] and Gomori trichrome [GT]). (C) Muscle fibers expressing myosin heavy chain (MyHC) type I (blue), type IIa (green) and type IIx (red). Fiber size variability affects all fiber types and there are numerous hybrids expressing both type IIa and IIx MyHC. (D) Small vacuolated muscle fibers and large fibers with regular intermyofibrillar network (NADH‐tetrazolium reductase [NADH‐TR]). (E) Lysosomal‐associated membrane protein 2 (LAMP2) immunohistochemistry showing increased number and size of lysosomes (arrows). (F) Many vacuolated fibers show p62+ protein aggregates (arrows). (G) Increased expression of microtubule‐associated protein 1A/1B‐light chain 3 (LC3) (arrows) (H) Mitochondrial adenosine triphosphate (ATP) synthase (mitochondrial ATP synthase membrane subunit c locus 3/subunit c of mitochondrial ATP synthase [ATP5MC3/SCMAS]) showing accumulation with the same distribution as LC3 (arrows), in addition to normal staining of mitochondria. (I) Increased deposition of autofluorescent (AF) material, mainly in muscle fibers (arrows). (Bar =50 μm).

**FIGURE 3:**
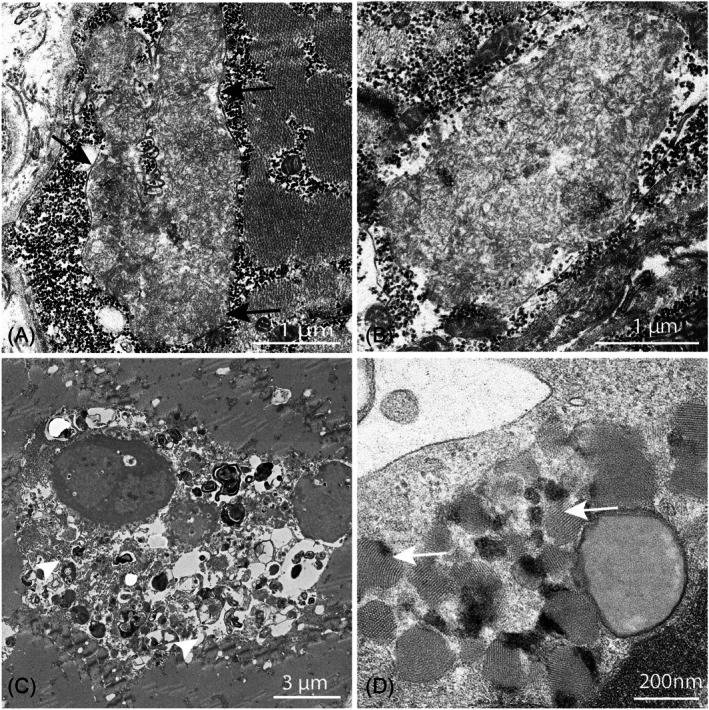
Electron microscopical analysis of skeletal muscle (A–C) and peripheral blood lymphocytes (PBLs) (D). (A, B) Storage of granular, curved and curvilinear material in lysosomes lined by a single membrane (arrows). (C) Autophagic vacuoles in a muscle fiber. (D) Fingerprint inclusions in PBLs (arrows).

The muscle histopathology is illustrated in Figures 2, 3, and S1–S17. The vastus lateralis muscle showed marked fiber size variability with hypertrophic and atrophic muscle fibers, interstitial fibrosis and fat tissue replacement (Figure 2A,B). Many small fibers and occasional large fibers showed rimmed vacuoles (Figure 2B). Fiber size variability involved both type 1 and type 2 fibers (Figure 2C). Many type 2 fibers were hybrids expressing both type IIa and IIx MyHC. No fiber type grouping or group atrophy was seen. Several small fibers expressed embryonic MyHC, indicating ongoing regeneration (not shown). The intermyofibrillar network appeared to be preserved in most of the fibers except in small, vacuolated fibers (Figure 2D). Glycogen was evenly distributed in most fibers, but small focal accumulations of periodic acid‐Schiff (PAS)‐positive material were observed in several fibers (Figure S5). There was marked proliferation and enlargement of lysosomes, which were immunohistochemically marked by LAMP2 (Figure 2E). Many of the vacuolated fibers contained protein aggregates marked by sequestosome1/p62 (Figure 2F).

The proteomic analysis showed upregulation of subunit c of mitochondrial adenosine triphosphate (ATP) synthase (SCMAS, *ATP5MC3* [mitochondrial ATP synthase membrane subunit c locus 3]), previously reported to be accumulated in lysosomes in several forms of NCL [21, 22]. Therefore, staining for ATP5MC3/SCMAS and microtubule‐associated protein 1A/1B‐light chain 3 (LC3), a marker of autophagy, was performed on serial sections. There was an apparent colocalization of ATP5MC3/SCMAS and LC3 (Figure 2G,H).

Analysis of autofluorescence in UV‐light showed markedly increased deposition of autofluorescent lipofuscin in muscle fibers and vessel walls (Figure 2I). Enzyme histochemical investigation of mitochondria (cytochrome c oxidase [COX], succinate dehydrogenase [SDH], and COX/SDH) did not show any major abnormalities (Figure S14). Immunohistochemical investigation of sarcolemma‐associated proteins (dystrophin, caveolin‐3, and laminin alpha 2‐chain) revealed normal staining of the sarcolemma and only occasional vacuoles or invaginations with sarcolemma‐like features (Figure S15–S17). Autophagic vacuoles, which were mainly seen in small fibers, did not show sarcolemma‐like features.

Electron microscopic investigation of muscle tissue revealed numerous single‐membrane‐bound inclusions consisting of granular, curved, and curvilinear material (Figure 3A,B) in addition to large and small autophagic vacuoles with various content, including debris with numerous myelin‐like membrane profiles (Figure 3C). No vacuoles with sarcolemma‐like features were identified by electron microscopy. PBLs showed fingerprint inclusions (Figure 3D).

### 3.3 Genetic analysis

**FIGURE 4:**
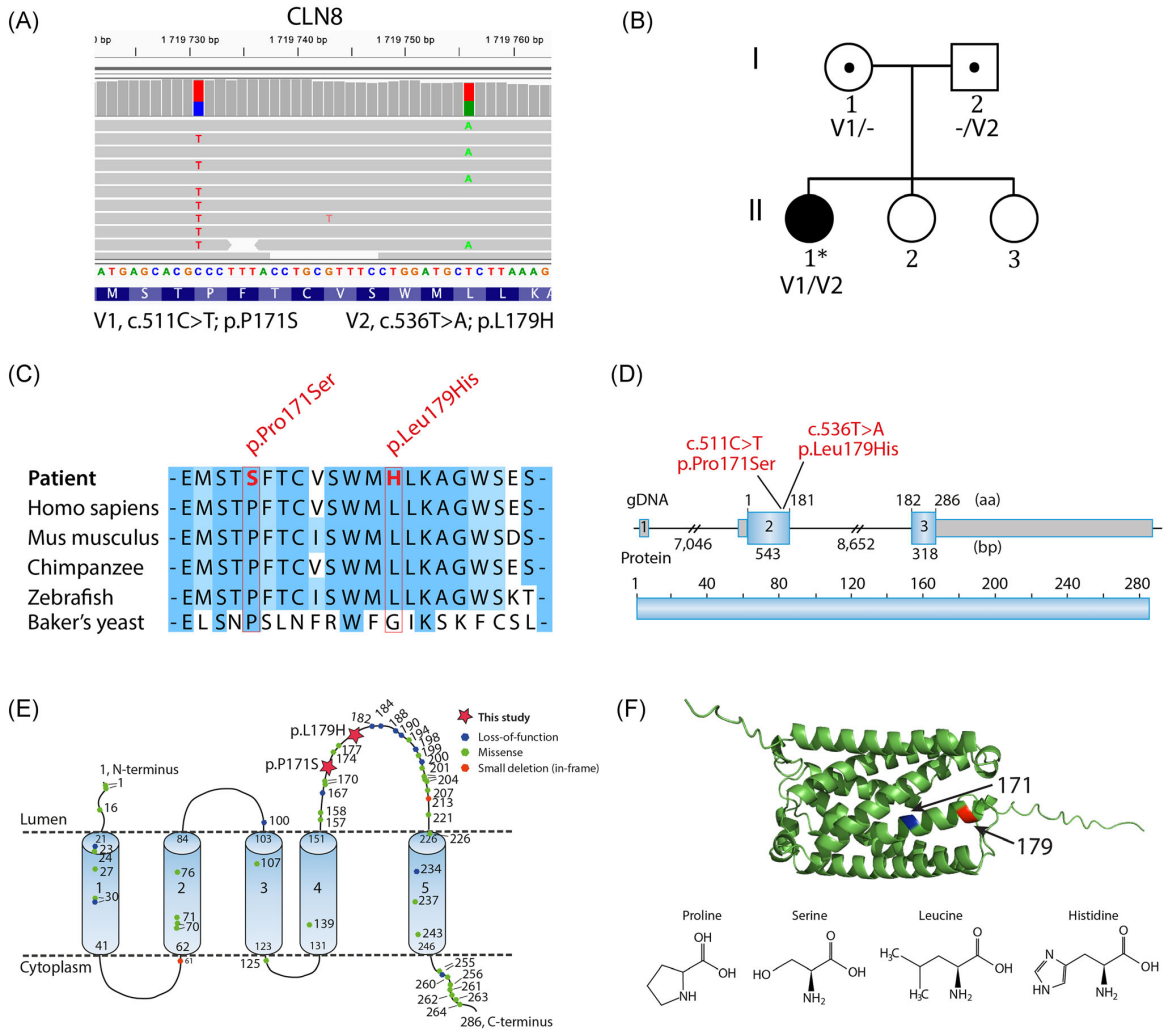
Molecular genetic analysis and protein modeling. (A) Visualization of the two variants identified in this study in the *CLN8* gene detected by WGS, c.511C>T; p.Pro171Ser and c.536T>A; p.Leu179His (NM_018941.4) (IGV software v2.18.4). (B) Pedigree of the family, the two identified variants were inherited from the mother and the father respectively. (C) At protein level the amino acid proline (P) at position 171 is replaced by a serine (S) (p.P171S) and at position 179 leucine (L) is replaced by a histidine (H) (p.L179H), both affected amino acids are highly conserved between species. (D) Schematic illustration of CLN8 at the genomic DNA (gDNA) and protein level. The patients' variants are located in exon 2. (E) Schematic illustration showing previously identified pathogenic variants in relation to the endoplasmic reticulum (ER). Variants identified in this study are marked in red and located in the ER luminal parts of the protein, close to several earlier reported variants. (F) Illustration of the stereo view of CLN8 protein with the amino acid proline at position 171 marked in blue and leucine at position 179 marked in red. This figure was generated using The PyMOL Molecular Graphics System, Version 1.7.2.1 Schrödinger, LLC. At the bottom, the structures of the involved amino acids are shown.

Genetic analysis identified two biallelic heterozygous missense variants in CLN8 (NM_018941.4), c.511C>T (p.Pro171Ser) and c.536T>A (p.Leu179His), inherited from the mother and father, respectively (Figure 4A,B). Neither variant has previously been reported as disease‐causing. In silico prediction algorithms supported pathogenicity: c.511C>T; p.Pro171Ser had a combined annotation‐dependent depletion (CADD) score of 26.7 and was absent from gnomAD, while c.536T>A; p.Leu179His had a CADD score of 28.1 and a gnomAD allele frequency of 0.0032% (Figure 4A–F). Both variants affect highly conserved amino acid residues (Figure 4C) and are located within a cytoplasmic region of the protein, near previously reported variants (Figure 4E) [23], within an alpha‐helix (Figure 4F).

### 3.4 Proteomic investigation

The proteomic analysis identified and quantified 4349 proteins in all samples, of which 70 proteins were significantly and differentially expressed in the patient compared to the control group (adjusted *p*‐value [FDR] <0.05): 51 proteins were increased and 19 were decreased (included in Table S3).

**FIGURE 5:**
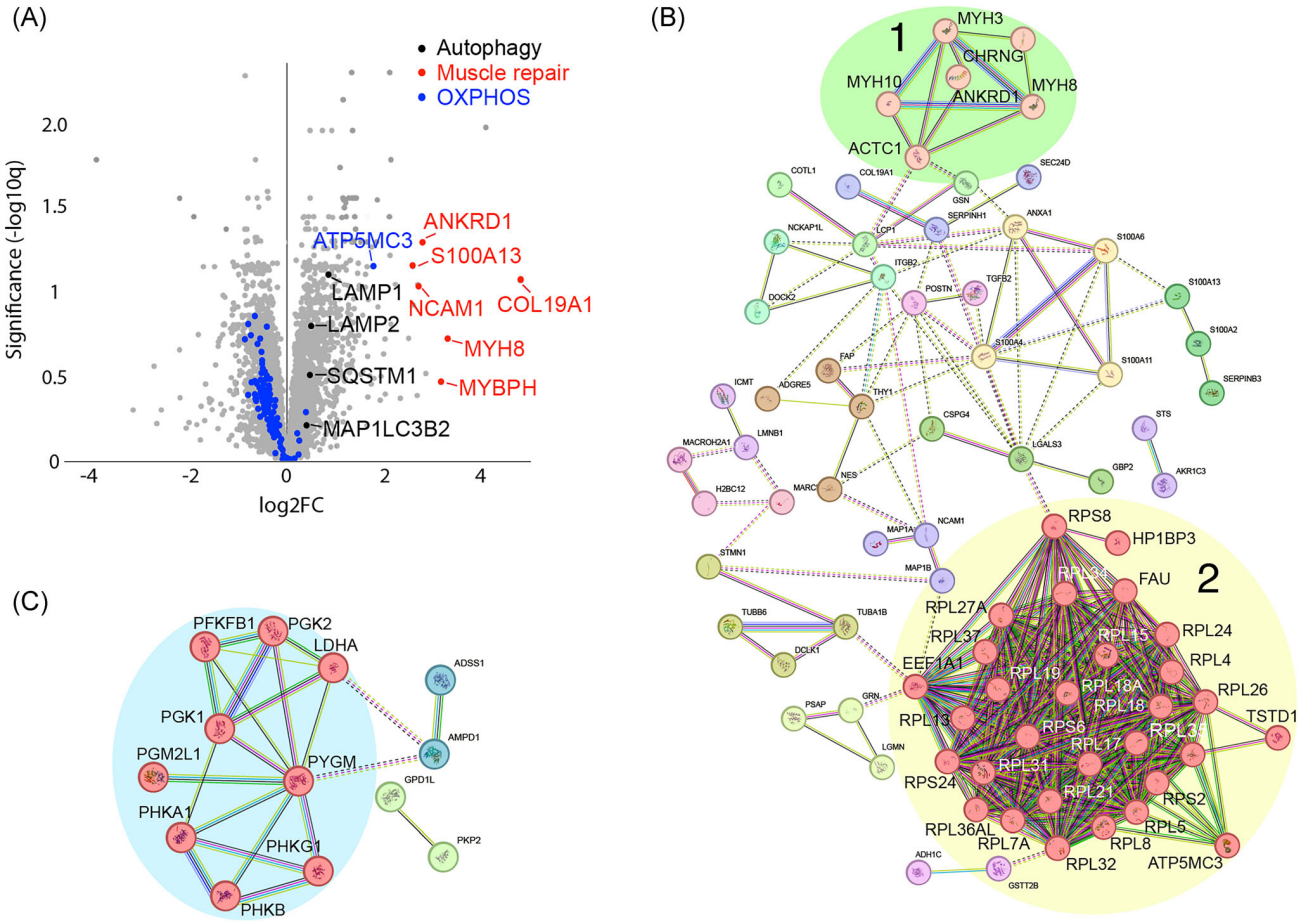
Proteomic analysis of dysregulated proteins. (A) Volcano plot showing upregulated proteins involved in muscle repair marked in red and autophagic markers marked in black. Most of the proteins involved in oxidative phosphorylation (OXPHOS) were downregulated (marked in blue) except mitochondrial adenosine triphosphate (ATP) synthase membrane subunit c locus 3/subunit c of mitochondrial ATP synthase (ATP5MC3/SCMAS), which was highly enriched. (B) From the STRING analysis, two clusters were detected among the upregulated proteins and highlighted: 1, proteins involved in muscle repair; 2, ribosome‐associated proteins. (C) From the STRING analysis, one cluster was detected among the downregulated proteins and highlighted: Proteins involved in glycogenolysis. (False discovery rate [FDR] = 0.2; log2 fold change <−1 or >1, 139 proteins).

Gene ontology (GO‐term) and pathway‐based in silico analyses of dysregulated proteins were performed to elucidate the biological processes and subcellular compartments affected in the patient. The overall proteomic profiling revealed several major alterations compared to normal muscle. These included signs of increased muscle repair, with upregulation of proteins involved in muscle regeneration and development (Figure 5A,B, cluster 1), as well as increased markers of autophagy and protein turnover, evidenced by augmented protein synthesis and reflected by the upregulation of ribosome‐associated proteins (Figure 5B, cluster 2). Conversely, among the downregulated proteins were those involved in oxidative phosphorylation (OXPHOS) [24], the process by which cells generate most of their ATP through electron transfer along the electron transport chain in the inner mitochondrial membrane, except for ATP5MC3/SCMAS that was upregulated (Figure 5A). In addition, proteins involved in glycogenolysis were downregulated (Figure 5C).

## 4 DISCUSSION

We describe a novel myopathy with autophagic vacuoles in an adult patient with epilepsy, muscle weakness, and neuropsychiatric symptoms, associated with novel compound heterozygous variants in *CLN8*. Muscle biopsy revealed characteristic features of lysosomal ceroid lipofuscin storage, including curvilinear‐like and autofluorescent material containing the mitochondrial ATP5MC3/SCMAS protein. In addition to ATP5MC3/SCMAS accumulation, proteomic analysis indicated increased muscle repair, enhanced markers of autophagy and protein turnover, and downregulation of proteins involved in oxidative phosphorylation and glycogenolysis.

The gene *CLN8* on chromosome 8 encodes an ER transmembrane protein involved in transfer of lysosomal enzymes [8]. Two major different clinical presentations are known to be associated with *CLN8*, EPMR [9, 10], and late‐infantile NCL (v‐LINCL) [11]. Other *CLN8* variants causing v‐LINCL have been reported in Italy [25], Israel [23], and Poland [26] among other countries, and a spectrum of *CLN8*‐associated phenotypes ranging from congenital NCL to Northern epilepsy has been proposed, with onset from congenital to adolescence [26]. *CLN8* has been associated both with fingerprint profiles and curvilinear bodies within lysosomes in skin biopsies and blood lymphocytes [27].

Storage of lipopigment with ultrastructural characteristics of ceroid lipofuscin in skeletal muscle of patients with NCLs has been reported [28], but among the different NCLs, autophagic vacuolar myopathy has been described as part of the clinical manifestations only in CLN3 [29, 30, 31, 32, 33]. In childhood forms of NCL, the myopathy may be subtle or overlooked in view of visual and cerebral problems. CLN3‐associated myopathy presents as a lysosomal vacuolar myopathy with rimmed vacuoles and membrane‐bound accumulations of autofluorescent lipopigment with curved or curvilinear and straight profiles [27, 28, 31, 34, 35]. The novel myopathy described in this report, associated with biallelic *CLN8* variants, shows striking similarities to CLN3‐associated myopathy. These similarities include the presence of autophagic vacuoles containing cellular debris and SQSTM1/p62 aggregates, as well as subsarcolemmal and intermyofibrillar intralysosomal aggregates of autofluorescent material with ultrastructural features, such as curvilinear profiles, consistent with ceroid lipofuscinosis.

Despite these morphological similarities, the underlying molecular etiology differs between CLN3 and CLN8. CLN3 is a lysosomal membrane protein, whereas CLN8 localizes primarily to the ER, with partial localization to the ER–Golgi intermediate compartment (ERGIC) [36]. CLN8 and CLN6 function as obligate partners in the recruitment of newly synthesized lysosomal enzymes in the ER, while the subsequent transfer of these enzymes to the Golgi apparatus is mediated exclusively by CLN8 [37].

A common downstream consequence of deficiency in these NCL‐associated proteins is thought to be impaired lysosomal function, leading to defective degradation of specific molecules and the subsequent accumulation of undigested proteins. One protein known to accumulate in several forms of NCL, including CLN3 and CLN8, is subunit c of mitochondrial ATP synthase, ATP5MC3/SCMAS [21, 22]. As one of three paralog genes encoding subunit c, *ATP5MC3* is a part of the last step of the oxidative phosphorylation and is critical for ATP synthesis. Our immunohistochemical and proteomic analyses revealed the accumulation of this protein in the patient's muscle, further supporting the diagnosis of ceroid lipofuscinosis. Notably, this accumulation of ATP5MC3/SCMAS contrasted with the downregulation of most other OXPHOS subunits, indicating impaired mitochondrial energy metabolism. In parallel, proteins involved in glycolysis were also reduced (Figure 5C), suggesting an overall deficit in muscle energy metabolism.

Among the differentially expressed proteins that were more abundant in the patient's muscle than in controls, several could be associated with ongoing muscle fiber regeneration (Figure 5B). These proteins are developmentally regulated and are typically expressed during early muscle development as well as during regeneration following necrosis of adult muscle fibers. These proteomic findings are consistent with the histopathological features of a degenerative–regenerative myopathy, including the presence of numerous regenerating muscle fibers expressing embryonic myosin heavy chain (MYH3).

Our patient differed clinically from previously described cases of CLN8 by adult‐onset and very slow disease progression. The episode of transient mental fatigue probably was an early symptom. While our patient had normal visual acuity, the macular pigmentations might consist of macrophages with pigment accumulation. Interestingly, photosensitivity at EEG has been described as an early feature of CLN2‐associated disease [38], and our patient's seizures were triggered by screen usage. In contrast to patients with EPMR and v‐LINCL, however, anti‐seizure medication was effective. Skin symptoms are rarely reported in NCL and might have been a coincidental finding. No skin biopsy was available for assessment.

The occurrence of affected PBLs, symptoms and signs from the central nervous system, and elevated liver enzymes suggesting hepatic damage indicate a systemic involvement and further support the diagnosis of a CLN8‐associated vacuolar myopathy in our patient. The histopathological evidence of a myopathy with autophagic vacuoles and accumulation of autofluorescent material and lysosomal storage of ATP5MC3/SCMAS was in line with the proteomic identification showing increased abundance of autophagy‐related markers including LAMP1, LAMP2, SQSTM1/p62, and LC3, as well as an increase of ATP5MC3/SCMAS (Figure 5A).

In conclusion, we describe a vacuolar myopathy with characteristic morphological features of lysosomal ceroid lipofuscin storage disease in muscle and lymphocytes that may occur as an unusual adult‐onset form of CLN8. Our data emphasize the importance of further evaluation in patients developing symptoms from multiple organ systems and show the value of muscle biopsy, electron microscopy of PBLs, and genetics to establish an NCL diagnosis.

## AUTHOR CONTRIBUTIONS

**Ulrika Lindgren**: Methodology, investigation, formal analysis, funding acquisition, visualization, project administration, writing—original draft, writing—review and editing. **Carola Hedberg‐Oldfors**: Conceptualization, methodology, investigation, formal analysis, funding acquisition, visualization, writing—original draft, writing—review and editing. **Kittichate Visuttijai**: Investigation, visualization, writing—review and editing. **Sara Nordström**: Investigation, writing—review and editing. **Hans Goebel**: Methodology, writing—review and editing. **Anders Oldfors**: Conceptualization, methodology, investigation, formal analysis, funding acquisition, visualization, writing—original draft, writing—review and editing.

## FUNDING INFORMATION

The study was financed by Swedish governmental funding of clinical research (ALF) to Carola Hedberg‐Oldfors and Anders Oldfors, the Swedish Research Council (No 2025‐03309 to Anders Oldfors), and the Research Fund for Neuromuscular Disorders in West Sweden to Ulrika Lindgren, Carola Hedberg‐Oldfors, and Anders Oldfors.

## CONFLICT OF INTEREST STATEMENT

The authors declare no conflict of interest.

## ETHICS STATEMENT

This study was approved by the Regional Ethics Committee at the University of Gothenburg, approval no. D390‐07 and conducted according to the Declaration of Helsinki. The patient gave written informed consent to participate.

## Supporting information


**Data S1.** Supporting Information.

## Data Availability

The data that support the findings are available from the corresponding author upon reasonable request.
