# Supplementary material for: A novel myopathy with autophagic vacuoles associated with biallelic variants in CLN8
Source: Brain Pathol. 2026 Jul 26:e70128. Online ahead of print. doi: 10.1111/bpa.70128 (PMC13402235; doi:10.1111/bpa.70128)
Supplement: Supplementary file 1 — Data S1. Supporting Information. [file BPA-9999-e70128-s001.pdf]

Supplementary data

A novel myopathy with autophagic vacuoles associated with biallelic variants in *CLN8*

Ulrika Lindgren[1,2,3], Carola Hedberg-Oldfors[1,3,4], Kittichate Visuttijai[1,2], Sara Nordström[1], Hans Goebel[5], Anders Oldfors[1,3]

Corresponding author
Ulrika Lindgren, ulrika.lindgren@gu.se
Department of Laboratory Medicine, Institute of Biomedicine, Sahlgrenska Academy, University of Gothenburg, Gothenburg, Sweden

Table S1. Antibodies used in immunohistochemistry of muscle tissue.

Antigen	Host (Clonality)	Dilution	Manufacturer (Clone, Catalogue Number)
**ATP synthase c**	Rabbit (mAb)	1:2000	Abcam (EPR13907, ab181243)
**Caveolin-3**	Mouse (mAb)	1:1000	Santa Cruz Biotechnology (A-3, sc-5310)
**C5b-9** (MAC)	Mouse (mAb)	1:50	Dako, Agilent (aE11, M0777)
**Dystrophin** (Dys-2, C-terminus)	Mouse (mAb)	1:100	Novocastra, Leica Biosystems (DY8/6C5, NCL-Dys2)
**LAMP-2** (CD107b)	Mouse (mAb)	1:300	SouthernBiotech (H4B4, 9840-01)
**LC3B** (MAP1LC3B)	Rabbit (pAb)	1:1000	Sigma-Aldrich (L7543)
**Merosin** (Laminin alpha 2 chain)	Mouse (mAb)	1:100	Novocastra, Leica Biosystems (Mer3/22B2, NCL-Merosin)
**Myosin heavy chain** (MYH3, embryonic)	Mouse (mAb)	1:20	Developmental Studies Hybridoma Bank (DSHB) (F1.652)
**Myosin heavy chain** (MYH8, fetal/neonatal)	Mouse (mAb)	1:20	Novocastra, Leica Biosystems (WB-MHCn, NCL-MHcn)
**Sequestosome 1** (SQSTM1/P62)	Mouse (mAb)	1:2000	Santa Cruz Biotechnology (SCBT) (D-3, sc-28359)

mAb, Monoclonal antibodies; pAb, Polyclonal antibodies; LAMP-2, lysosomal-associated membrane protein 2; LC3B, Microtubule-associated protein 1 light chain 3B; MAC, membrane attack complex

Table S2. Antibodies used in immunofluorescence for myofiber typing.

**Antigen** (Isotype/Conjugation)	**Host** (Clonality)	**Dilution**	**Manufacturer** (Clone, Catalogue Number)
Primary antibodies			
**MyHC Type I** (MYH7, IgG2b)	Mouse (mAb)	1:50	Developmental Studies Hybridoma Bank (DSHB) (BA-D5)
**MyHC Type IIA** (MYH2, IgG1)	Mouse (mAb)	1:50	Developmental Studies Hybridoma Bank (DSHB) (SC-71)
**MyHC Type IIX** (MYH1, IgM)	Mouse (mAb)	1:10	Developmental Studies Hybridoma Bank (DSHB) (6H1)
**Heparan Sulfate Proteoglycan (Perlecan)** (IgG2aκ)	Rat (mAb)	1:200	Merck (A7L6, MAB1948P)
Secondary antibodies			
**Mouse IgG1** (Alexa Fluor 488)	Goat	1:200	ThermoFisher Scientific (A-21121)
**Mouse IgM (H)** (Alexa Fluor 647)	Goat	1:200	ThermoFisher Scientific (A-21238)
**Mouse IgG2b** (Brilliant Violet 421)	Goat	1:200	Jackson ImmunoResearch (115-675-207)
**Rat IgG (H+L)** (Alexa Fluor 568)	Goat	1:200	ThermoFisher Scientific (A-11077)

MyHC: Myosin Heavy chain; HRP: Horseradish peroxidase

Table S3. 139 up- and down-regulated proteins were included in the STRING analysis (CLN8).

	Gene symbol	Descriptiom	Log2FC	FDR
Upregulated	COL19A1	collagen type XIX alpha 1 chain	4.79	0.08
	MYH3	myosin heavy chain 3	4.08	0.01
	MYH8	myosin heavy chain 8	3.29	0.19
	ANKRD1	ankyrin repeat domain 1	2.77	0.05
	NCAM1	neural cell adhesion molecule 1	2.68	0.09
	TMT1B	thiol methyltransferase 1B	2.59	0.12
	S100A13	S100 calcium binding protein A13	2.58	0.07
	S100A2	S100 calcium binding protein A2	2.50	0.18
	ACTC1	actin alpha cardiac muscle 1	2.23	0.09
	SP140L	SP140 nuclear body protein like	2.14	0.02
	TNFAIP8L3	TNF alpha induced protein 8 like 3	2.13	0.15
	S100A6	S100 calcium binding protein A6	2.11	0.06
	DCLK1	doublecortin like kinase 1	2.11	0.00
	TGFB2	transforming growth factor beta 2	2.10	0.04
	AKR1C3	aldo-keto reductase family 1 member C3	2.01	0.19
	H2BC12	H2B clustered histone 12	1.87	0.12
	TSTD1	thiosulfate sulfur transferase like domain containing protein 1	1.86	0.03
	NES	nestin	1.84	0.05
	ATP5MC3	ATP synthase membrane subunit c locus 3	1.76	0.07
	TUBB6	tubulin beta 6 class V	1.74	0.05
	S100A4	S100 calcium binding protein A4	1.67	0.11
	H4C1	H4 clustered histone 1	1.66	0.04
	FAU	FAU ubiquitin like and ribosomal protein S30 fusion	1.65	0.07
	PSAP	prosaposin	1.64	0.05
	RPL3	ribosomal protein L3	1.61	0.08
	ITGB2	integrin subunit beta 2	1.61	0.09
	GSTT2	glutathione S-transferase, theta 2	1.60	0.20
	THY1	Thy-1 cell surface antigen	1.56	0.13
	PTGFRN	prostaglandin F2 receptor inhibitor	1.51	0.09
	CHRNG	cholinergic receptor nicotinic gamma subunit	1.51	0.05
	SH3BP1	SH3 domain binding protein 1	1.49	0.09
	S100A11	S100 calcium binding protein A11	1.49	0.09
	TP53I3	tumor protein p53 inducible protein 3	1.49	0.09
	MAP1A	microtubule associated protein 1A	1.48	0.09
	FOLR2	folate receptor beta	1.47	0.18
	POSTN	periostin	1.46	0.07
	EEF1A1	eukaryotic translation elongation factor 1 alpha 1	1.45	0.08
	RPL7A	ribosomal protein L7a	1.42	0.03
	RPL34	ribosomal protein L34	1.41	0.04
	MRI1	methylthioribose-1-phosphate isomerase 1	1.41	0.19
	RPS24	ribosomal protein S24	1.39	0.04
	RPL31	ribosomal protein L31	1.39	0.05
	BID	BH3 interacting domain death agonist	1.39	0.18
	MLF2	myeloid leukemia factor 2	1.38	0.03
	MEGF10	multiple EGF like domains 10	1.38	0.12
	LCP1	lymphocyte cytosolic protein 1	1.37	0.11
	ANXA1	annexin A1	1.37	0.15
	CLIC4	chloride intracellular channel 4	1.35	0.05
	ADGRE5	adhesion G protein-coupled receptor E5	1.35	0.01
	LMNB1	lamin B1	1.34	0.03
	RPL8	ribosomal protein L8	1.34	0.05
	RPL26	ribosomal protein L26	1.33	0.00
	TPSB2	tryptase beta 2	1.31	0.17
	MXRA7	matrix remodeling associated 7	1.31	0.13
	RPL5	ribosomal protein L5	1.31	0.06
	SERPINH1	serpin family H member 1	1.30	0.18
	NCKAP1L	NCK associated protein 1 like	1.30	0.15
	LIMS4	LIM zinc finger domain containing 4	1.29	0.18
	LGMN	legumain	1.29	0.06
	FAP	fibroblast activation protein alpha	1.28	0.15
	MAP1B	microtubule associated protein 1B	1.28	0.13
	RPL37	ribosomal protein L37	1.27	0.20
	STMN1	stathmin 1	1.27	0.03
	RPL35	ribosomal protein L35	1.27	0.12
	SCPEP1	serine carboxypeptidase 1	1.27	0.12
	NUCB2	nucleobindin 2	1.26	0.20
	GSN	gelsolin	1.26	0.09
	MAGED2	MAGE family member D2	1.24	0.13
	ARMCX1	armadillo repeat containing X-linked 1	1.24	0.18
	RPL4	ribosomal protein L4	1.23	0.10
	DOCK2	dedicator of cytokinesis 2	1.22	0.07
	RPL27A	ribosomal protein L27a	1.22	0.04
	RPL36AL	ribosomal protein L36a like	1.22	0.07

	RPS6	ribosomal protein S6	1.21	0.03
	RPL18A	ribosomal protein L18a	1.19	0.05
	LGALS3	galectin 3	1.18	0.09
	RPL15	ribosomal protein L15	1.18	0.03
	RPL13	ribosomal protein L13	1.17	0.03
	COTL1	coactosin like F-actin binding protein 1	1.16	0.16
	RPL24	ribosomal protein L24	1.16	0.07
	RPS8	ribosomal protein S8	1.15	0.01
	RPS2	ribosomal protein S2	1.15	0.03
	RRAD	RRAD. Ras related glycolysis inhibitor and calcium regulator	1.15	0.18
	PREPL	prolyl endopeptidase like	1.14	0.15
	MYO1D	myosin ID	1.13	0.07
	RPL21	ribosomal protein L21	1.13	0.04
	CLIC1	chloride intracellular channel 1	1.11	0.12
	HNMT	histamine N-methyltransferase	1.11	0.15
	MACROH2A1	macroH2A.1 histone	1.10	0.14
	RPL18	ribosomal protein L18	1.09	0.02
	RNASET2	ribonuclease T2	1.09	0.11
	ABLIM1	actin binding LIM protein 1	1.09	0.13
	ICMT	isoprenylcysteine carboxyl methyltransferase	1.06	0.08
	CSPG4	chondroitin sulfate proteoglycan 4	1.06	0.07
	HP1BP3	heterochromatin protein 1 binding protein 3	1.06	0.06
	STS	steroid sulfatase	1.06	0.10
	GBP2	guanylate binding protein 2	1.05	0.10
	SEC24D	SEC24 homolog D. COPII coat complex component	1.05	0.20
	CXCL14	C-X-C motif chemokine ligand 14	1.04	0.18
	RPL17	ribosomal protein L17	1.04	0.14
	MYH10	myosin heavy chain 10	1.04	0.07
	RPL19	ribosomal protein L19	1.03	0.07
	GRN	granulin precursor	1.03	0.17
	MARCKS	myristoylated alanine rich protein kinase C substrate	1.03	0.13
	TUBA1B	tubulin alpha 1b	1.03	0.16
	ADH1C	alcohol dehydrogenase 1C (class I), gamma polypeptide	1.03	0.13
	RPL32	ribosomal protein L32	1.02	0.07
	SERPINB3	serpin family B member 3	1.02	0.16
	GANC	glucosidase alpha. neutral C	1.00	0.12
Downregulated	ERAP2	endoplasmic reticulum aminopeptidase 2	-3.90	0.02
	AQP4	aquaporin 4	-2.19	0.03
	MYLK2	myosin light chain kinase 2	-1.90	0.04
	PKP2	plakophilin 2	-1.81	0.18
	ART3	ADP-ribosyltransferase 3	-1.71	0.07
	GATD3	glutamine amidotransferase class 1 domain containing protein 3A	-1.48	0.07
	NIPSNAP3B	nipsnap homolog 3B	-1.40	0.19
	BDH1	3-hydroxybutyrate dehydrogenase 1	-1.37	0.18
	AMPD1	adenosine monophosphate deaminase 1	-1.31	0.18
	ASB14	ankyrin repeat and SOCS box containing 14	-1.30	0.10
	PMM2	phosphomannomutase 2	-1.30	0.15
	PHKG1	phosphorylase kinase catalytic subunit gamma 1	-1.28	0.15
	PHKA1	phosphorylase kinase regulatory subunit alpha 1	-1.27	0.15
	ADSS1	adenylosuccinate synthase 1	-1.26	0.11
	RRN3	RRN3 homolog. RNA polymerase I transcription factor	-1.24	0.04
	PFKFB1	6-phosphofructo-2-kinase/fructose-2,6-biphosphatase 1	-1.15	0.16
	PHKB	phosphorylase kinase regulatory subunit beta	-1.12	0.17
	STXBP5L	syntaxin binding protein 5L	-1.12	0.13
	LDHA	lactate dehydrogenase A	-1.11	0.12
	SVIP	small VCP interacting protein	-1.10	0.07
	PNLIPRP3	pancreatic lipase related protein 3	-1.09	0.15
	ATP2B2	ATPase plasma membrane Ca2+ transporting 2	-1.09	0.11
	SELENOW	selenoprotein W	-1.08	0.12
	NDST2	N-deacetylase and N-sulfotransferase 2	-1.07	0.07
	PYGM	glycogen phosphorylase. muscle associated	-1.05	0.16
	PGK1	phosphoglycerate kinase 1	-1.04	0.16
	PGK2	phosphoglycerate kinase 2	-1.02	0.15
	PGM2L1	phosphoglucomutase 2 like 1	-1.02	0.07
	MDN1	midasin AAA ATPase 1	-1.01	0.16
	GPD1L	glycerol-3-phosphate dehydrogenase 1 like	-1.00	0.12

Gene symbols are included for the encoded protein; Log2FC < -1 or >1; FDR = 0.2.

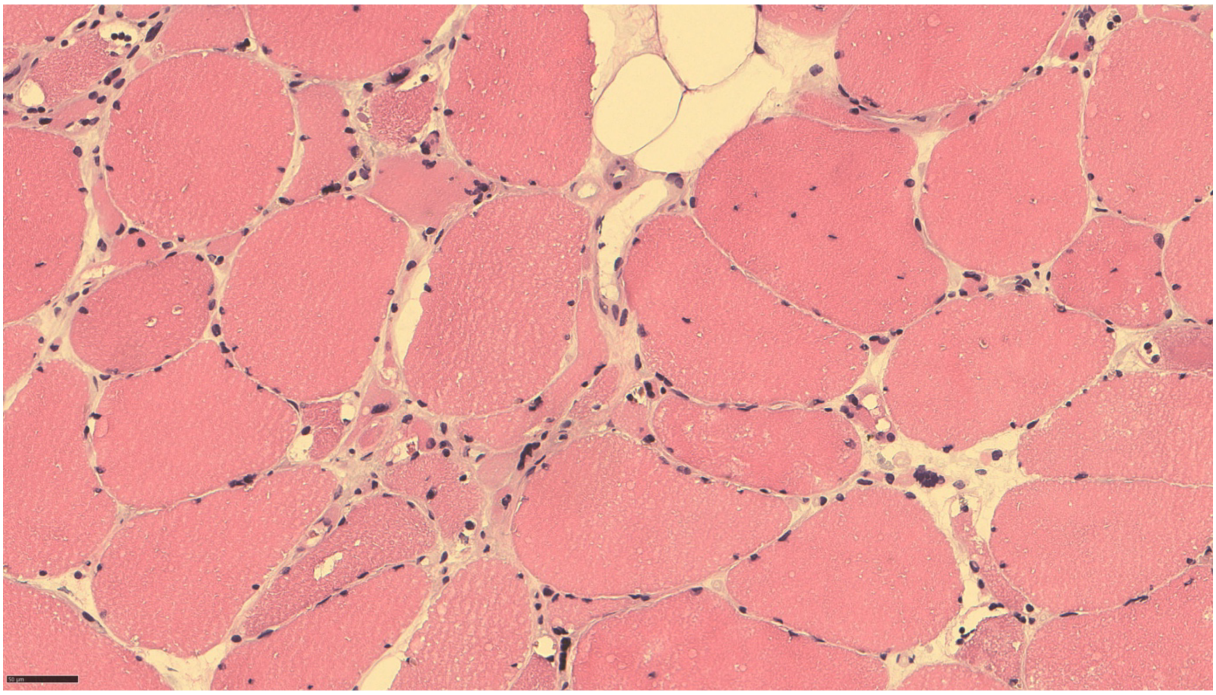

Figure S1. Muscle biopsy from the patient. Hematoxylin and eosin.
Scale bar = 50 µm

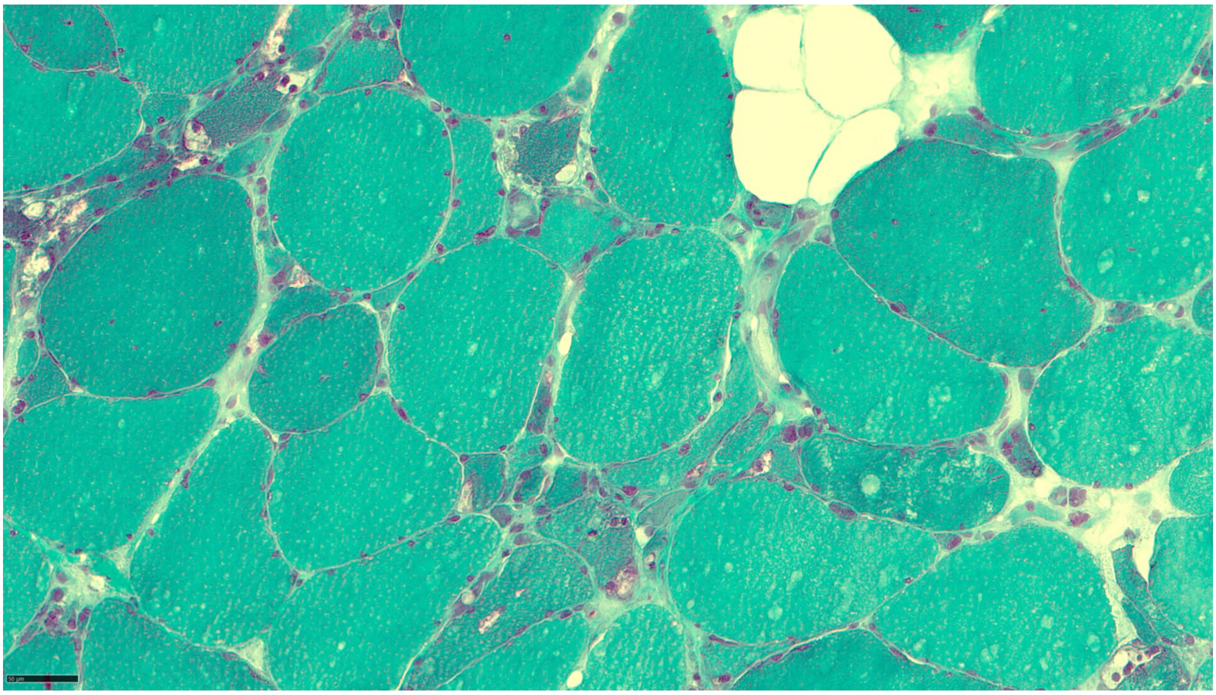

Figure S2. Muscle biopsy from the patient. Gomori trichrome.
Scale bar = 50 µm

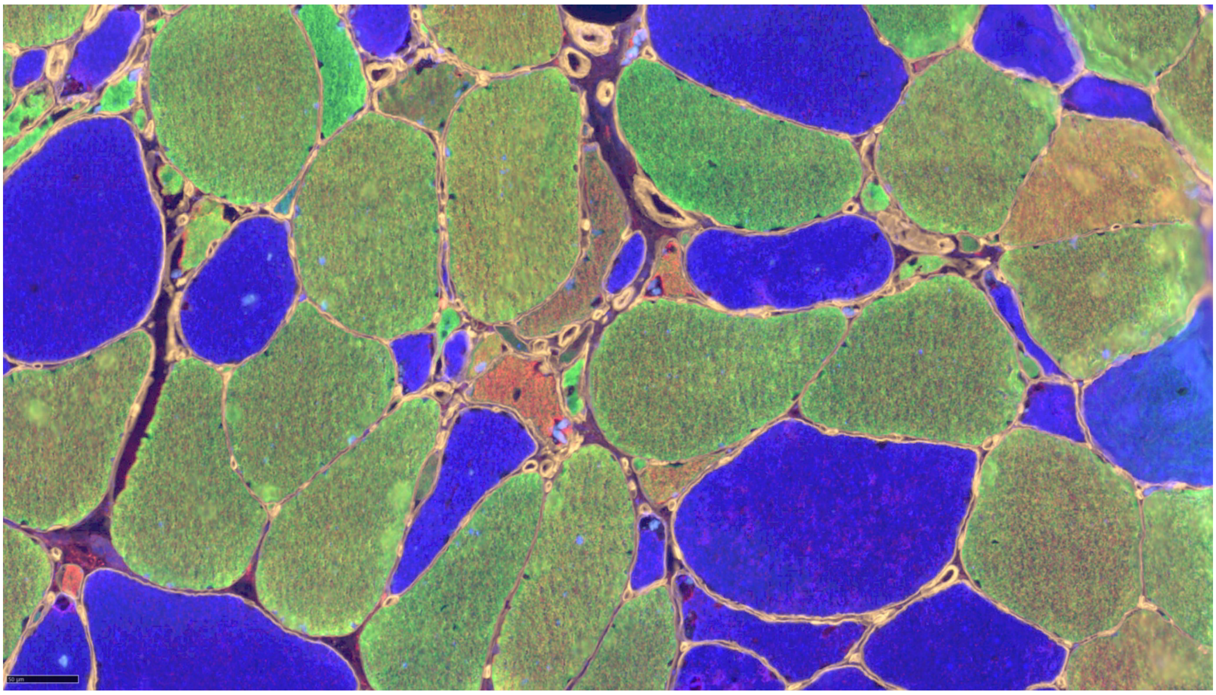

Figure S3. Muscle biopsy from the patient. Muscle fiber typing by immunofluorescence. Myosin heavy chain (MyHC) type I (blue), type IIa (green) and type IIx (red).
Scale bar = 50 µm

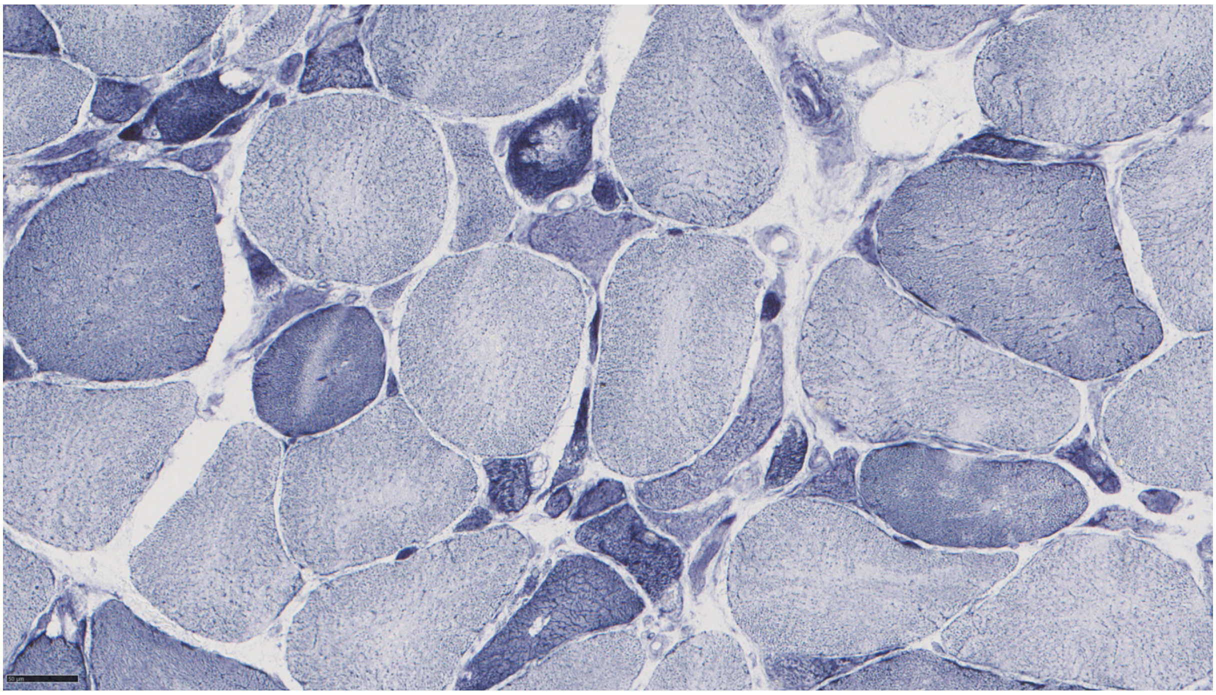

Figure S4. Muscle biopsy from the patient. NADH-TR.
Scale bar = 50 µm

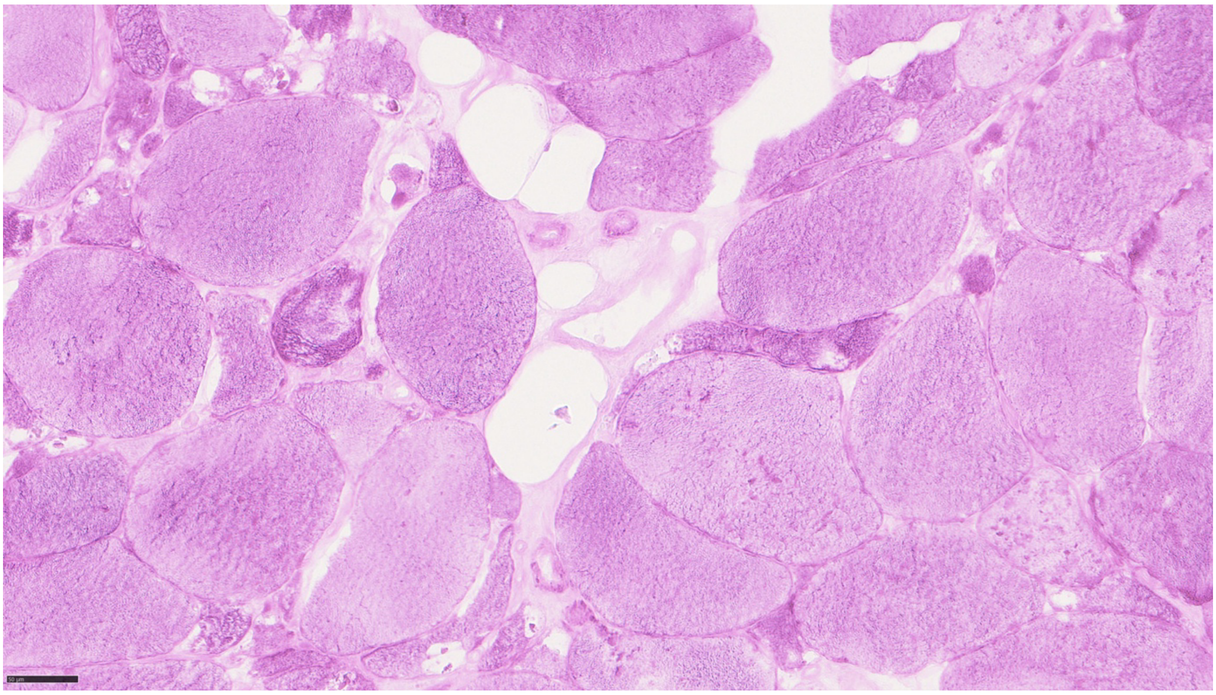

Figure S5. Muscle biopsy from the patient. Periodic acid-Schiff (PAS) stain.
Scale bar = 50 μm

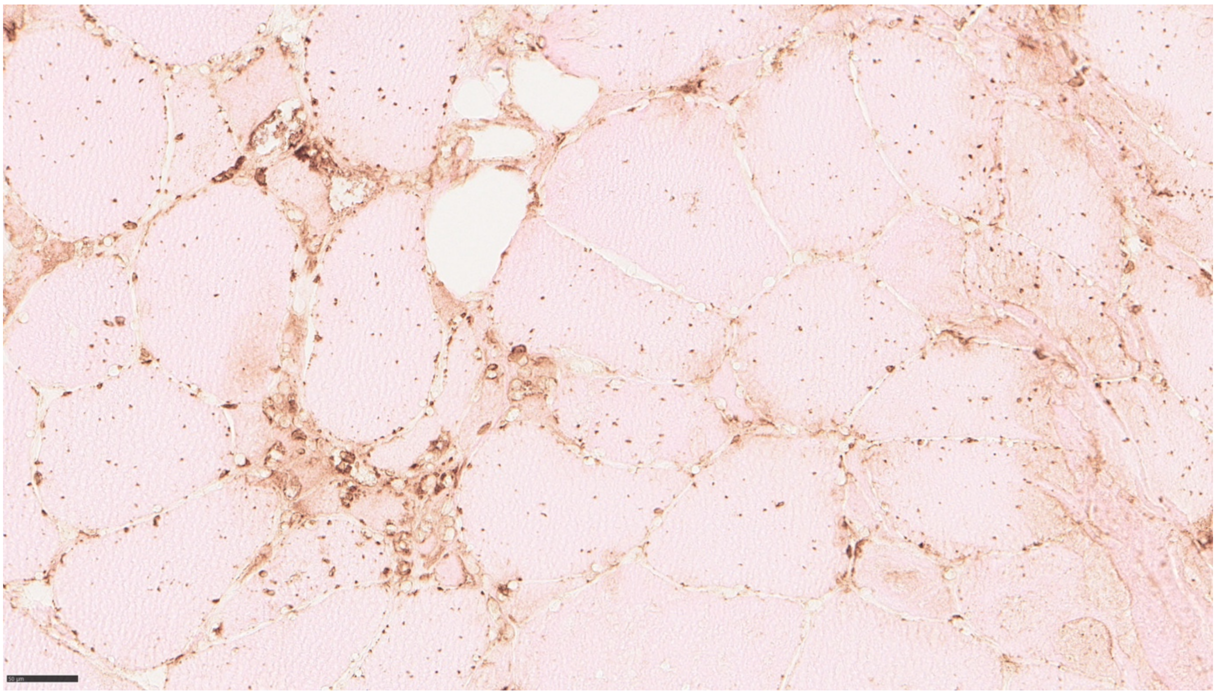

Figure S6. Muscle biopsy from the patient. LAMP2 immunohistochemistry.
Scale bar = 50 μm

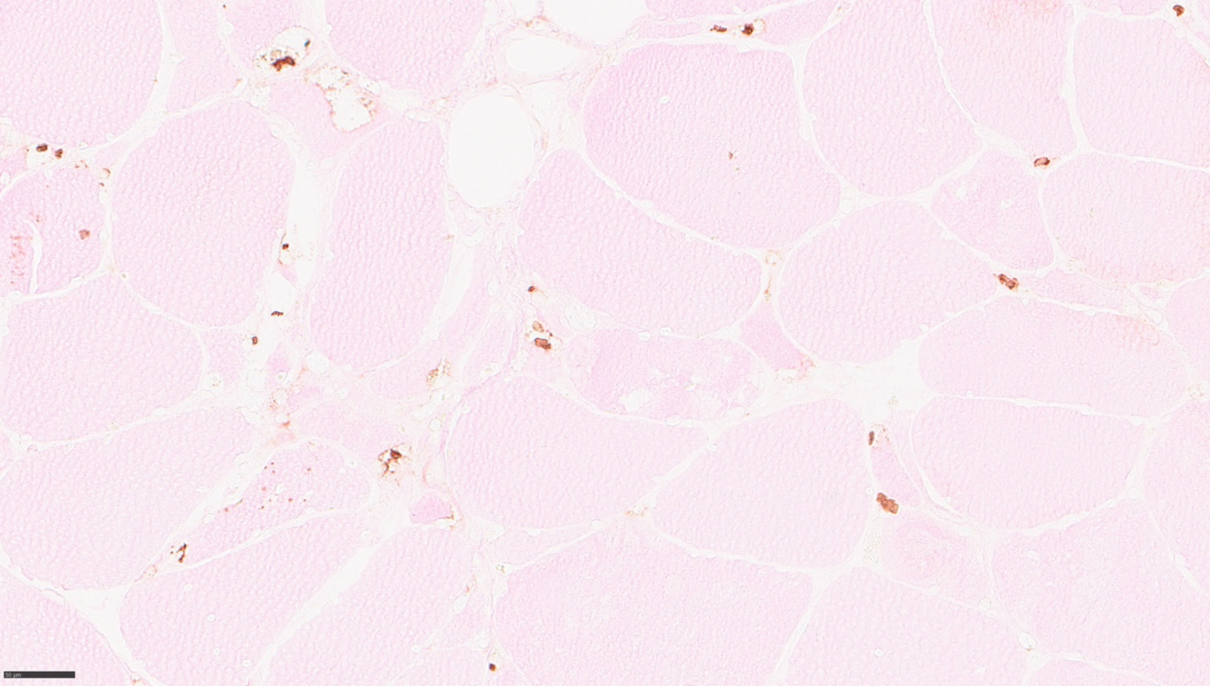

Figure S7. Muscle biopsy from the patient. Sequestosome1/p62 immunohistochemistry. Scale bar = 50 µm

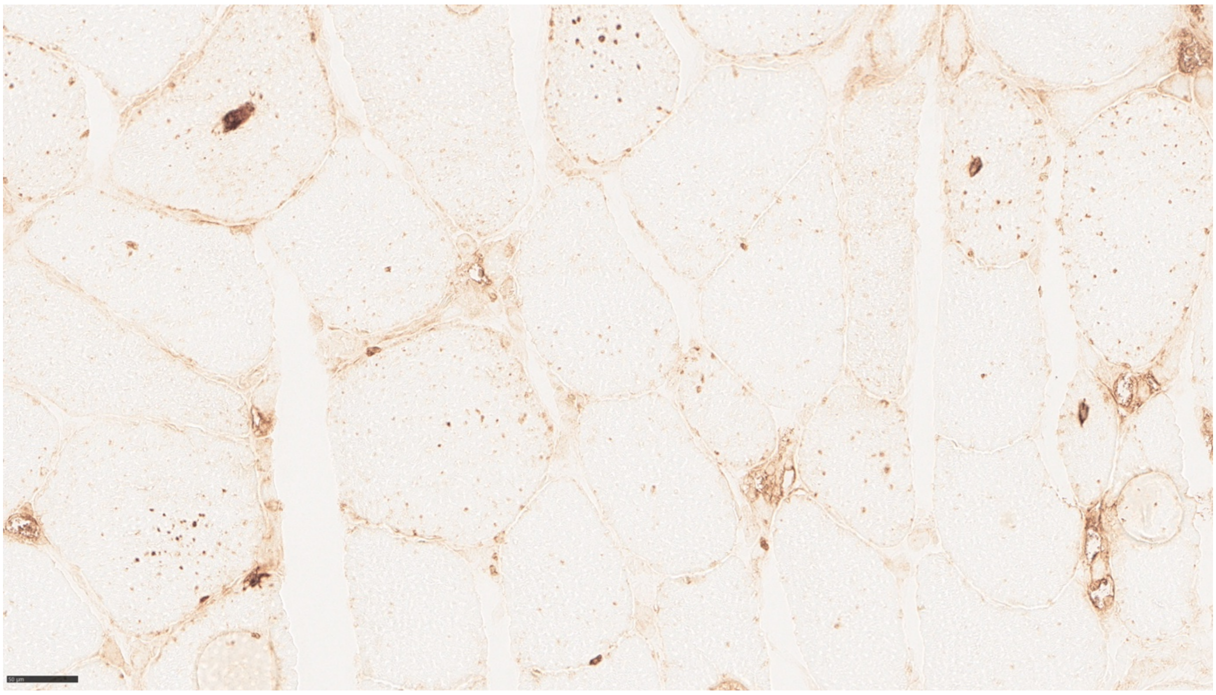

Figure S8. Muscle biopsy from the patient. LC3 immunohistochemistry.
Scale bar = 50 μm

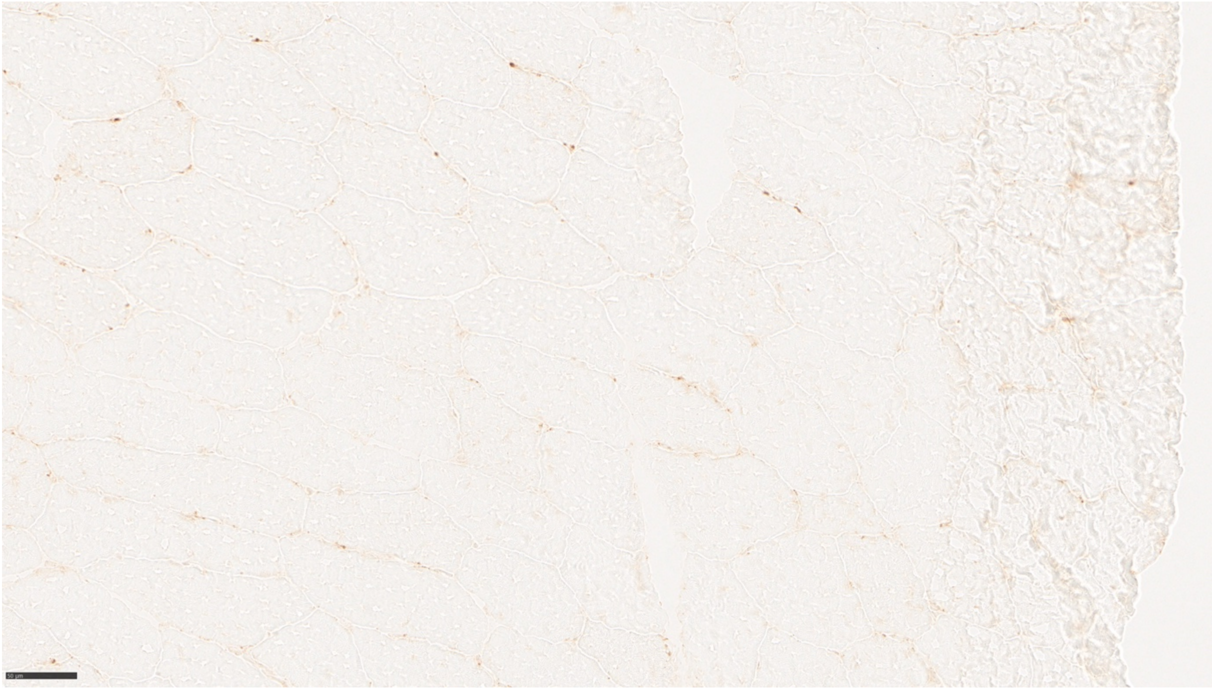

Figure S9. Muscle biopsy from normal control. LC3 immunohistochemistry.
Scale bar = 50 μm

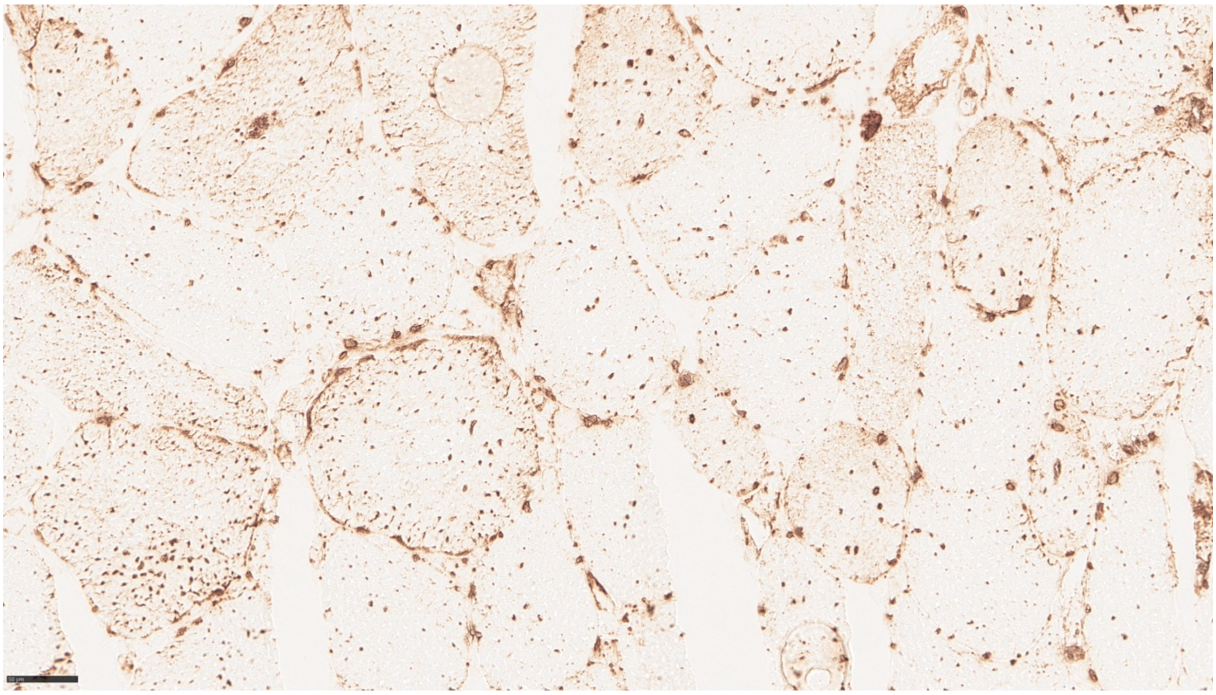

Figure S10. Muscle biopsy from the patient. ATP5MC3/SCMAS immunohistochemistry, showing mitochondrial localization in addition to larger deposits associated with accumulation in LC3 positive autophagosomes (see Figure S7).
Scale bar = 50 µm

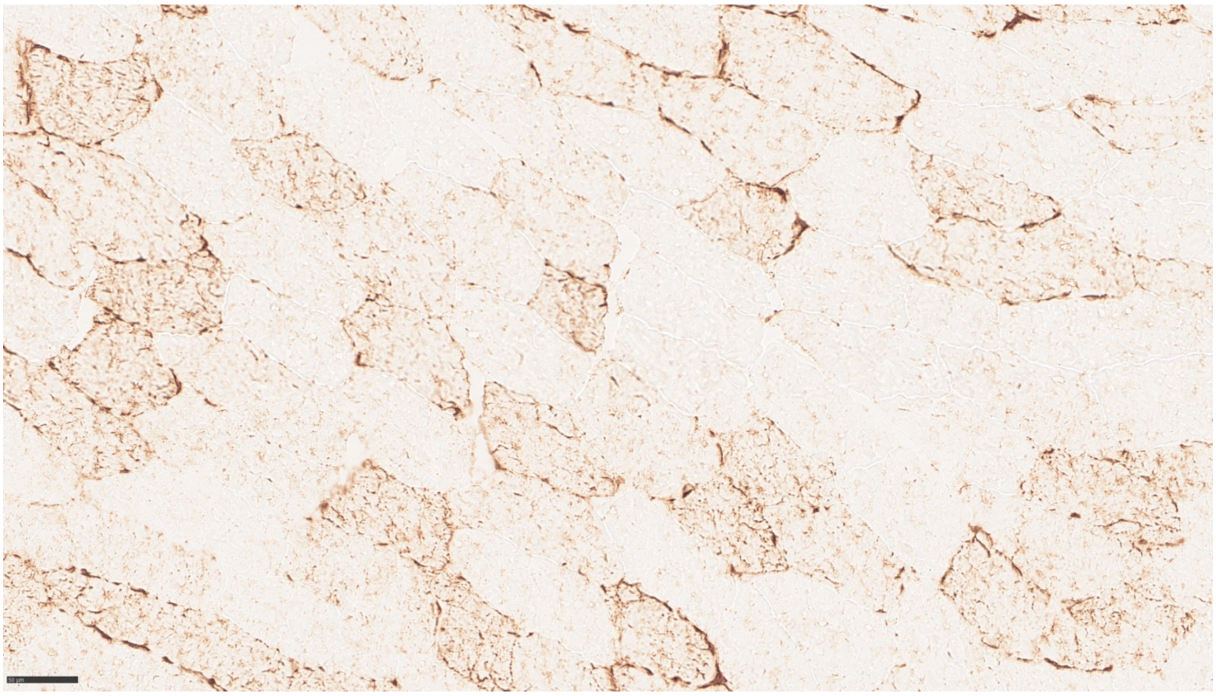

Figure S11. Muscle biopsy from normal control. ATP5MC3/SCMAS immunohistochemistry, showing mitochondrial localization.
Scale bar = 50 µm

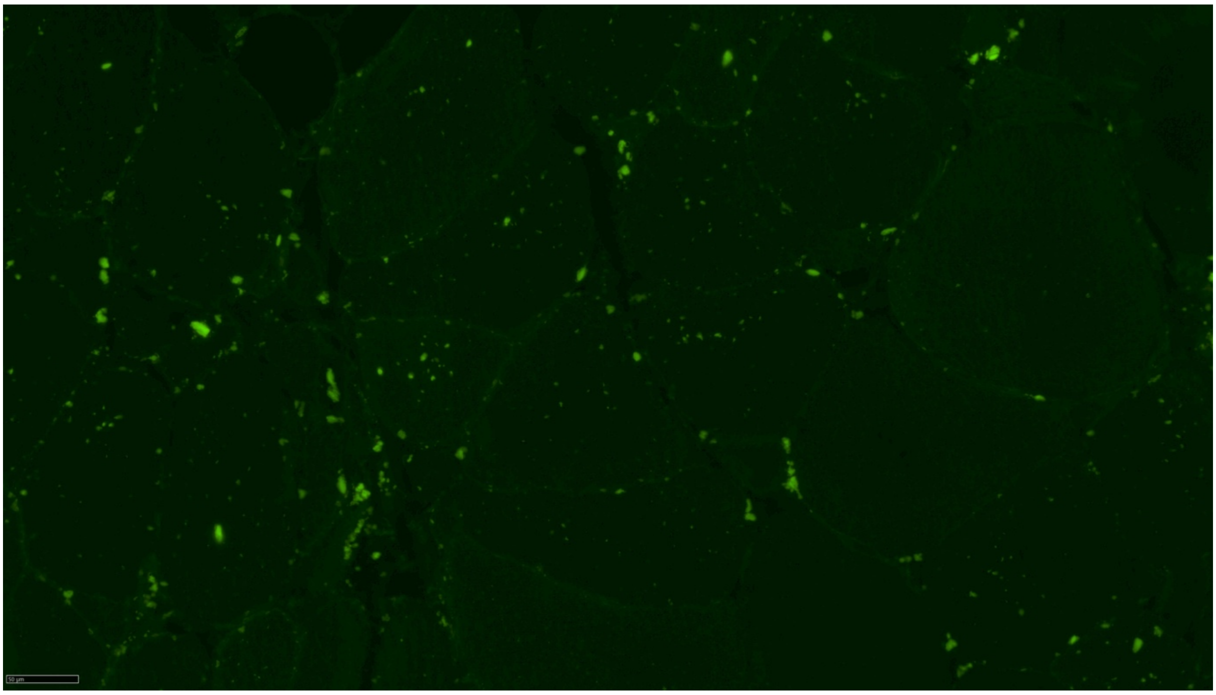

Figure S12. Muscle biopsy from the patient. Autofluorescence, unstained section.
Scale bar = 50 μm

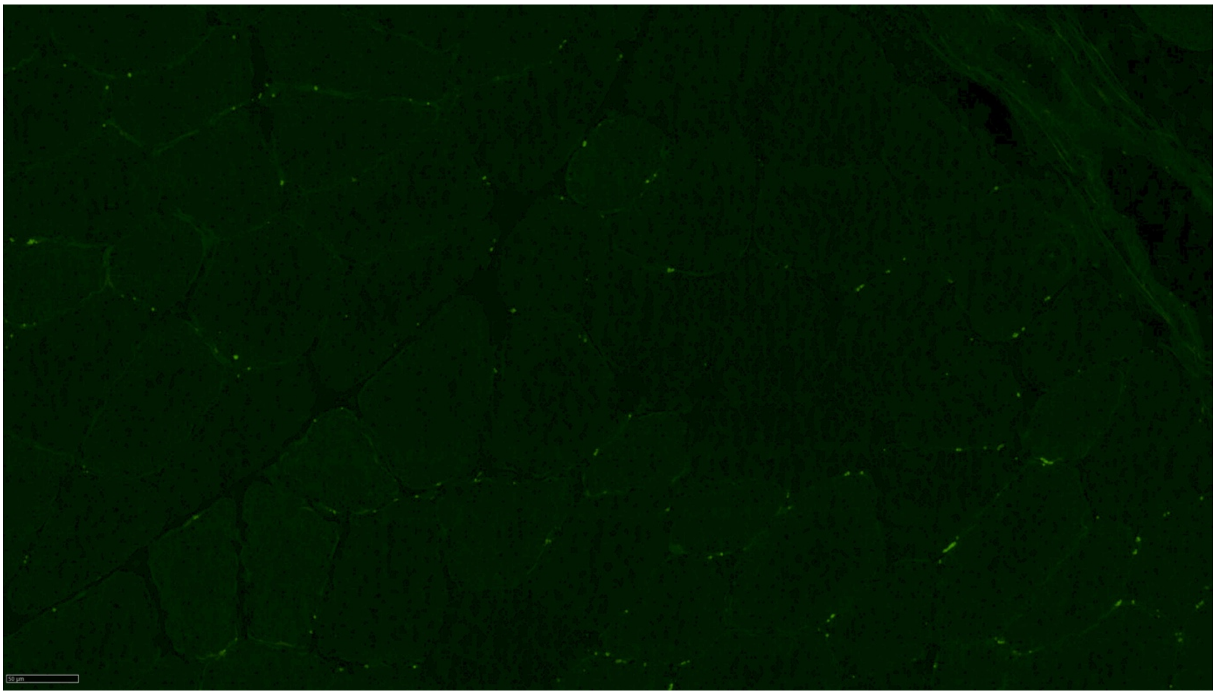

Figure S13. Muscle biopsy from normal control. Autofluorescence, unstained section.
Scale bar = 50 μm

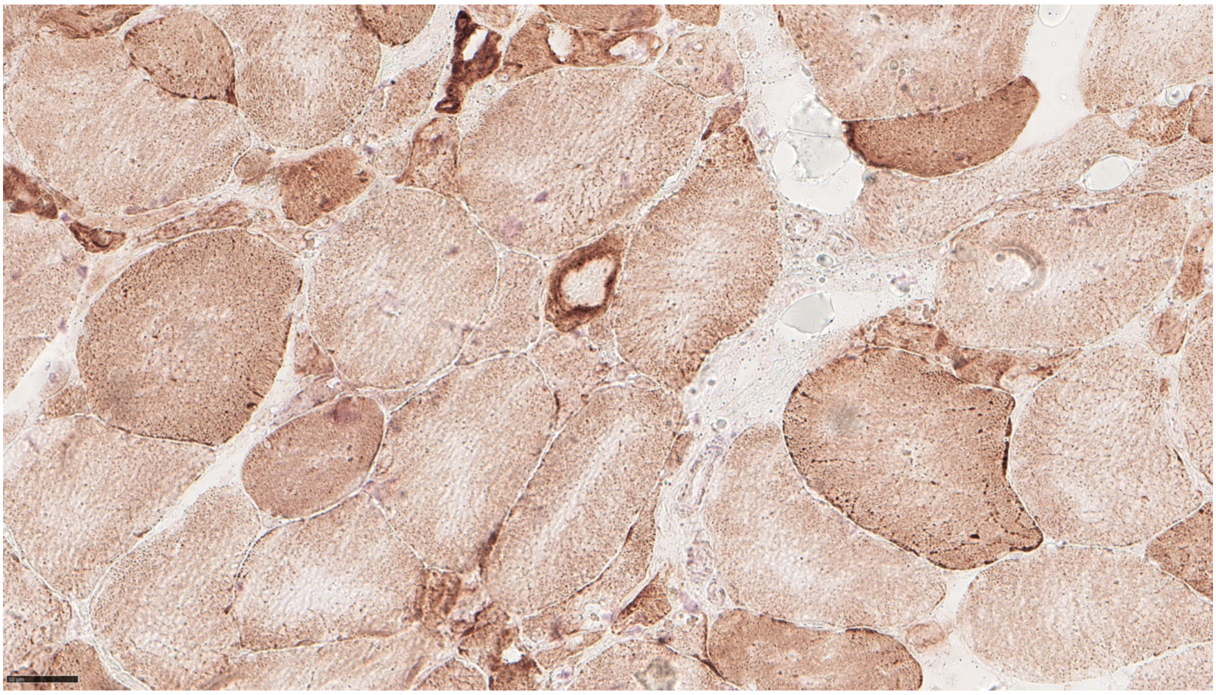

Figure S14. Muscle biopsy from the patient. Cytochrome c oxidase/Succinate dehydrogenase. Scale bar = 50 µm

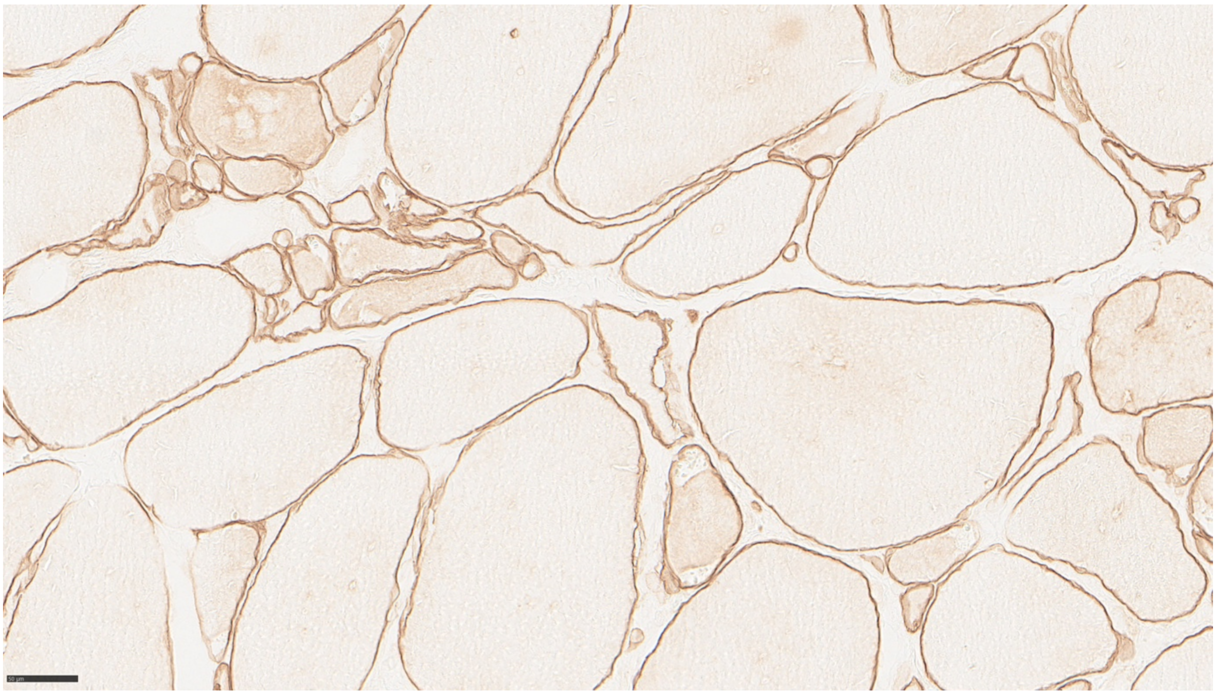

Figure S15. Muscle biopsy from the patient. Dystrophin (DYS2) immunohistochemistry. Scale bar = 50 µm

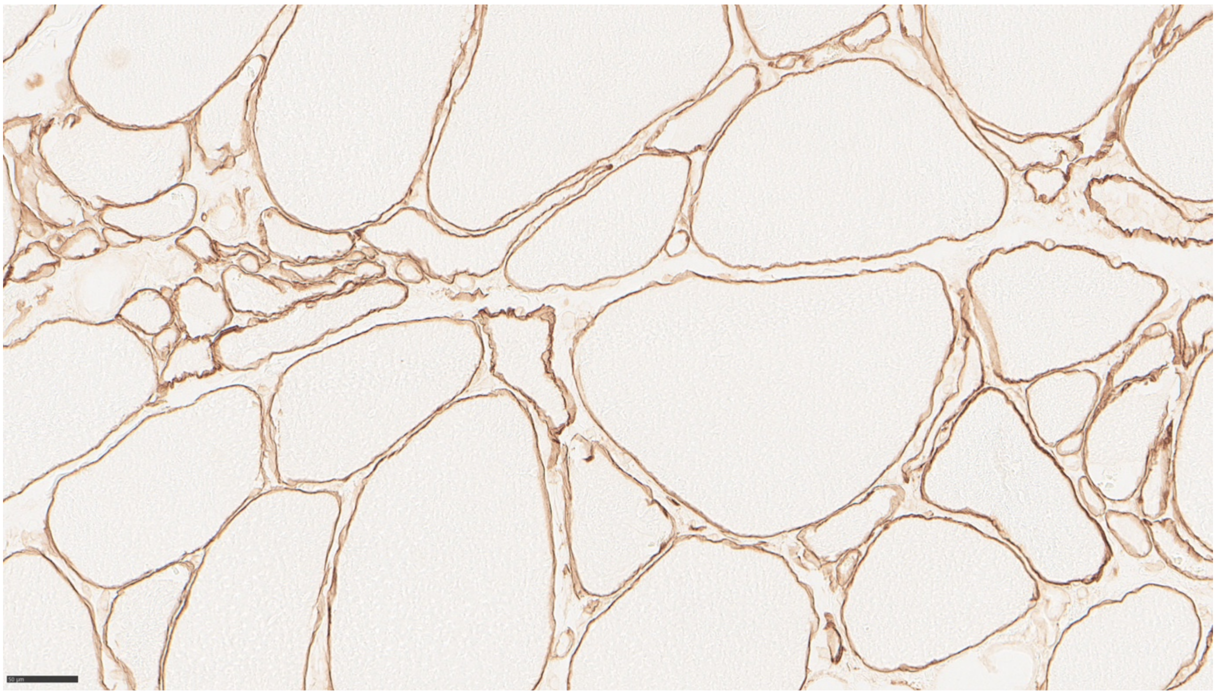

Figure S16. Muscle biopsy from the patient. Laminin alpha 2-chain immunohistochemistry.
Scale bar = 50 µm

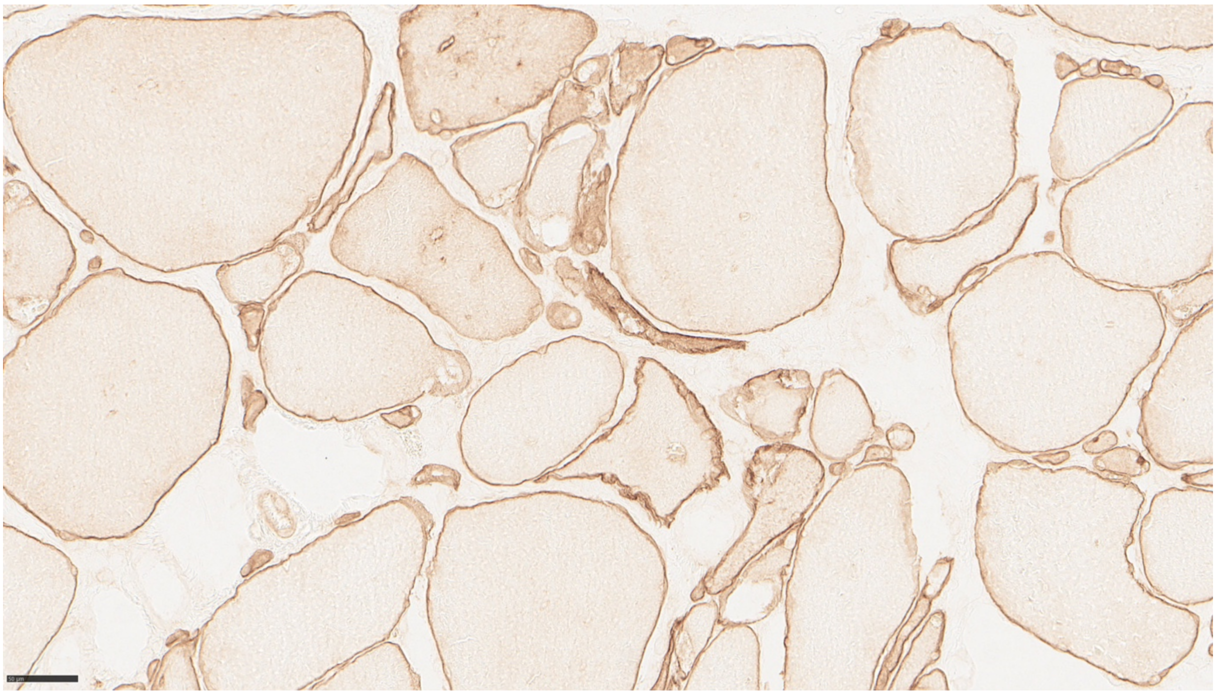

Figure S17. Muscle biopsy from the patient. Caveolin-3 immunohistochemistry.
Scale bar = 50 µm
